# Dynamics of Bacterial Communities during the Ripening Process of Different Croatian Cheese Types Derived from Raw Ewe's Milk Cheeses

**DOI:** 10.1371/journal.pone.0080734

**Published:** 2013-11-20

**Authors:** Mirna Mrkonjić Fuka, Stefanie Wallisch, Marion Engel, Gerhard Welzl, Jasmina Havranek, Michael Schloter

**Affiliations:** 1 Department Microbiology, Faculty of Agriculture, University of Zagreb, Zagreb, Croatia; 2 Research Unit for Environmental Genomics, Helmholtz Zentrum München, Neuherberg, Germany; 3 Department of Dairy Science, Faculty of Agriculture, University of Zagreb, Zagreb, Croatia; Charité-University Medicine Berlin, Germany

## Abstract

Microbial communities play an important role in cheese ripening and determine the flavor and taste of different cheese types to a large extent. However, under adverse conditions human pathogens may colonize cheese samples during ripening and may thus cause severe outbreaks of diarrhoea and other diseases. Therefore in the present study we investigated the bacterial community structure of three raw ewe's milk cheese types, which are produced without the application of starter cultures during ripening from two production sites based on fingerprinting in combination with next generation sequencing of 16S rRNA gene amplicons. Overall a surprisingly high diversity was found in the analyzed samples and overall up to 213 OTU_97_ could be assigned. 20 of the major OTUs were present in all samples and include mostly lactic acid bacteria (LAB), mainly *Lactococcus*, and *Enterococcus* species. Abundance and diversity of these genera differed to a large extent between the 3 investigated cheese types and in response to the ripening process. Also a large number of non LAB genera could be identified based on phylogenetic alignments including mainly *Enterobacteriaceae* and *Staphylococcacae*. Some species belonging to these two families could be clearly assigned to species which are known as potential human pathogens like *Staphylococcus saprophyticus* or *Salmonella* spp. However, during cheese ripening their abundance was reduced. The bacterial genera, namely *Lactobacillus*, *Streptococcus, Leuconostoc*, *Bifidobacterium*, *Brevibacterium*, *Corynebacterium, Clostridium, Staphylococcus, Thermoanerobacterium, E. coli, Hafnia, Pseudomonas, Janthinobacterium, Petrotoga, Kosmotoga, Megasphaera, Macrococcus, Mannheimia*, *Aerococcus, Vagococcus, Weissella* and *Pediococcus* were identified at a relative low level and only in selected samples. Overall the microbial composition of the used milk and the management of the production units determined the bacterial community composition for all cheese types to a large extend, also at the late time points of cheese ripening.

## Introduction

Cheeses of raw ewe's milk are generally characterized by a stronger taste and a richer flavor compared to cheeses made from pasteurized milk [1], [2]. The unique aroma of each cheese variety made from raw ewe's milk is a consequence of complex microbial metabolic activities. However, the absence of pasteurization can also support the growth of undesirable microbes and increase the likelihood of pathogen surviving in the “ready to eat” cheese matrix [3]. Croatian raw ewe's milk cheeses are hard cheeses that are produced by traditional techniques without pasteurization and application of starter cultures, and they are characterized by an aging process of 90–120 days. This aging time is rather long and the low pH and water activity (a_w_) of ripened ewe's milk cheeses usually does not support the growth of pathogens if the cheeses are completely mature [4]. However due to more and more commercial pressure, the ripening times are shortened and it has become market practice to sell the cheese as soon as possible. Therefore a close monitoring of bacterial communities and a reliable identification of beneficial as well as potential pathogenic bacteria is crucial in order to maintain the quality and safety of cheese mainly produced from raw ewe's milk [5], [6]. Traditionally, the occurrence of microorganisms in cheese has been studied by culture-based methods. Nevertheless, it is well recognized that these methods can not reveal bacterial richness and evenness since cheese may harbor complex consortia of microorganisms of which only a minor part can be easily isolated [7]. Moreover, many culture media lack selectivity [8], [9] and often many bacteria (non culturable cells) cannot grow in a given selective medium [10], [11]. Ampe et al. [12] showed that at least 25–50% of the active microbial population in a food matrix could not be cultured in vitro although ‘culturable’ microorganisms generally predominate in such habitats [13]. In comparison to culture dependent methods, culture independent methods based on DNA/RNA analysis are less time consuming, more sensitive, more specific and more accurate. However, they have not often been used to study microbial diversity in food matrices, except for fingerprinting approaches based on 16S rRNA gene amplification flowed by denaturing gradient gel electrophoresis (DGGE) or clone library construction [6], [9], [14], [15], [16].

Recent advances in the sequencing technology, such as the development of pyrosequencing, has been described as promising, it is supposed to be less labor intensive and more informative in characterizing the microbial diversity in given habitats, compared to either culture depending methods or molecular fingerprinting approaches [17], [18], [19], [20]. Pyrosequencing provides a fast and massive sequencing approach, which can provide thousands of unique sequences in food and food related matrices [17], [20]. This output is much higher compared to what can be obtained using traditional cloning techniques and enables also the characterization of the rare biosphere in an environment [20], [21], [22]. Pyrosequencing thus is a promising tool to study microbial complexity of food and will expand our understanding of the microbial community structure more comprehensively than other molecular approaches currently in use.

Therefore the aim of the present study was to analyze shifts in bacterial diversity during the ripening of artisanal raw ewe's milk cheeses manufactured at two different production sites using a deep sequencing approach of amplicon libraries based on the 16S rRNA gene. In addition to that, the generated amplicons were used to perform a fingerprint analysis based on T-RFLP to assess potential heterogeneity levels of replicate samples before sequencing.

## Materials and Methods

### Cheese sampling

Three types of cheese, Cheese A (Istrian cheese), Cheese B (Krcki cheese) and Cheese C (Paski cheese) were investigated in this study. Each of the three types of cheese has been produced by two farm makers (F1 and F2), located on the peninsula of Istria in accordance with the traditional cheese making procedure using raw, full-cream milk from sheep. [23], [24], [25] For each cheese type independent batches of milk were used resulting in 6 batches of milk (2 farms×3 cheese types). From each cheese type per farm 3 replicates (based on the same batch of milk) were studied, resulting in 18 selected cheeses that were analyzed at each time point in the frame of this study. Cheeses samples were taken by drilling at day zero (0d), at 45 (45d) and at 90 (90d) days of ripening. Thus overall 54 samples (3 cheese types×2 farms×3 replicates×3 time points) were obtained. All samples were transported to the laboratory immediately after sampling in frozen condition and stored at −80°C until DNA extraction. Total water content of the ripened cheese samples ranged between 25 (cheese A) and 37% (cheese C); the pH values were in the range of 4.9 and 5.2; salt content varied between 2.1 (cheese B) and 4.9% (cheese C) and fat content between 30 (cheese C) and 39% (cheese B).

### DNA extraction from cheese samples

The total DNA was extracted and purified from 10 g of cheese after homogenization in a sterile physiological solution using a Stomacher (BagMixer® 400) for 3 minutes at 230 rpm. 10 ml of the homogenate were centrifuged at 3500×g for 10 min prior to the DNA extraction. The supernatant was discarding and the fat layer, which was at the top of liquid, was mechanically removed by a sterile filter paper. DNA was extracted by Maxwell Tissue DNA Purification Kit (Promega, Madison, USA). The DNA extracted was quantified using Quant-iT Picro Green dsDNA Reagent and Kits (Molecular Probes, Invitrogen). The integrity of the DNA extracted from the cheese samples was confirmed by running DNA extracts on 2% agarose gel.

### Terminal restriction fragment length polymorphisms (T-RFLP) analysis

Universal eubacterial primers 927f (5′-AAACTYAAAKGAATTGACGG-3′, [26] *E. coli* position 908–927) and 630r (5′-CAKAAAGGAGGTGATCC-3′, [27] *E. coli* position 1529 to 1545) were used for amplifying a 637 bp long region of the 16S rRNA gene. The forward primer was labeled with 6-Carboxyfluorescein (6-FAM). PCR reactions were performed in 25 µl (final volume) mixtures, containing 1X PCR buffer, 1.8 mM MgCl_2_ (Invitrogen, USA), 0.04 mM dNTPs (Fermentas, Germany), 0.3% BSA, 0.2 pmol of each primer, 0.025 U/µl Taq polymerase (Invitrogen, USA) and 40 ng template. Amplification was performed under the following conditions: initial denaturation at 95°C for 5 min, followed by 22 cycles of denaturation at 94°C for 1 min, annealing at 50°C for 1 min and elongation at 72°C for 1 min. Final extension was at 72°C for 10 min. PCR products were purified with NucleoSpin gel and PCR clean up (Machery Nagel, Germany). To obtain the highest number and most even distribution of terminal restriction fragments (T-RFs), a computer simulated analysis was utilized in order to select the most appropriate restriction enzymes for comparing the structure of cheese bacterial communities prior to the analysis. Therefore an *in-silico* PCR was performed with Genomatix v4.2 (http://www.genomatix.de/, Genomatix Software GmbH, Germany) and *in-silico* digestions of amplicons were made by a repk tool (http://rocaplab.ocean.washington.edu/tools/repk, [28]. After comparing the variety of theoretical digests' outputs, the restriction enzyme MaeIII (Roche, Switzerland) provided the best results for assessing the cheese bacterial community dominated by lactic acid bacteria (data not shown). To confirm the results obtained from the database analysis and to optimize digestion conditions for a reliable comparison of cheese bacterial communities, four bacterial species commonly described as typical for cheese derived from raw ewe's milk including *Pseudomonas fluorescens*, *Enterococcus faecium*, *E. faecalis*, *Lactobacillus* spp. and *Lactococcus lactis* were tested (data not shown). Thus, the obtained PCR products were digested using the restriction enzyme MaeIII (Roche) as recommended by the manufacturer and purified using the NucleoSpin gel and PCR clean up (Macherey Nagel). For the separation, MapMarker 1000 labeled with 6-Carboxyl-X-Rhodamine ROX (Eurogentec, Germany) and diluted 1∶600 with Hi-Di™ Formamide (ABI, Germany) was added to each sample as an internal standard. Fragments were separated by an ABI 3730 sequencer and the data was analyzed using the GeneMapper v4.0 software (ABI, USA) and T-REX v1.12 (http://trex.biohpc.org/, [29]).

The statistical data analysis of the T-RFLP profiles was done using R v2.12.1 (http://www.R-project.org/), both by creating binary matrices whereby the presence or absence of individual T-RFs was scored, and by calculating the relative abundance of T-RFs normalized by the total height of the respective T-RF patterns. The respective dendrograms were constructed using unweighted pair-group methods using arithmetic averages (UPGMA) analysis and Jaccard distance measure.

### Barcoded pyrosequencing and sequence processing

The same universal eubacterial primers (927f and 630r) as for T-RFLP analysis were used for 454 pyrosequencing. To identify samples after sequencing, unique Multiplex Identifiers followed by a four base library key and an adaptor site were added to the primers. HotStarTaq Plus Master Mix Kit (Qiagen, Germany) was used for PCR under the same conditions as for T-RFLP. The PCR products were purified by QIAquick PCR Purification Kit (Qiagen, Germany) and quantified by Quant-iT Picro Green dsDNA Reagent and Kits (Molecular Probes, Invitrogen). All amplicon products from different samples were mixed in equal volumes. In preparation for sequencing, the size and concentration of DNA fragments were accurately measured by the BioAnalyzer 2100 microfluidics device (Agilent, USA) using a DNA7500 lab chip (Agilent, USA). The samples were diluted to 10^9^ molecules/µl and stored at −20°C until further analysis. The 16S rRNA genes were sequenced with the 454 GS FLX Titanium Series from Roche, Germany (Roche, USA). A sample containing 10^6^ molecules/µl of double-stranded DNA with a size of 637 bp was combined with 9.6 million DNA capture beads, and then amplified by emulsion PCR. After bead recovery and bead enrichment, the bead-attached DNA was denatured with NaOH, and the sequencing primers were annealed. A two-region sequencing run (including amplicons generated using the forward respectively the reversed primer) was performed on a quarter of PicoTiterPlate (PTP). Based on the T-RFLP analysis, one of the three replicates from each of the analyzed samples was applied, thus eighteen (n = 18) samples were analyzed by 454 pyrosequencing in this study. All FLX related procedures were performed following the manufacturer's manual of the Genome Sequencer FLX System (Roche, USA). The sequences obtained in this study were uploaded and made available through the NCBI database under the number: KF358776 - KF358986.

### Sequence treatment and data analysis

The raw sequences were processed with the software MOTHUR v. 1.14.0 [30] to reduce sequencing errors. Therefore each flowgram was separated according to each barcode and primer, and the sequence was capped at a minimum length of 200bp. Thereby one and two mismatches were allowed for barcode and primers, respectively. Subsequently, the data was de-noised and then primers, barcodes and homopolymers were removed. For analyzing the sequencing data with reverse primers, also the reverse complement was considered. In the following step, the totally identical sequences were pooled and an alignment was generated using a dataset of the SILVA database provided by MOTHUR. For the forward and reverse sequences, both datasets were run against a SILVA-compatible alignment derived from a sequence collection of the Genomes online database [31]. This method provided less false-positive hits than running the chimera check against the corresponding data of the dataset itself. In a final step, any sequences that origin from mitochondria were excluded. A distance matrix was calculated from the high quality aligned sequences, and operational taxonomical units (OTUs; 90–100% sequence similarity) were assigned by using the furthest neighbour clustering algorithm. As 0% dissimilarity in sequences will provide a dramatic overestimation of the species present in a sample [22], 3% dissimilarity was used in oder to obtain an accurate estimation of the species present in a respective sample. OTUs defined by a 3% distance level were submitted to the RDP II database containing 164,517 almost full-length 16S rRNA sequences using an 80% confidence threshold to obtain the taxonomic assignment and the relative abundance of the different bacterial groups from genus to phylum [32]. NCBI (http://www.ncbi.nlm.nih.gov/) was used to convert the sample sequence data to bacterial phylogeny. The species showing the best match to representative OTUs sequences was assigned to the query sequence, and if more species showed the same best score, the one with the highest count in the top 15 was selected.

### Statistical analysis

From the output of the MOTHUR software, a distance matrix was separately created for the forward and reverse reads for statistical analyses. The clusters based upon dissimilarity of 3%, served as OTUs for generating predictive rarefaction models and for calculating the richness and diversity indices. Data were analyzed with statistical models using the R project software (v2.15.1, http://www.r-project.org). To compare the overall bacterial community of three different cheese types (Cheese A, B and C) at different ripening stages (0, 45 and 90d), OTU-based analyses were performed. Therefore, pyrosequencing reads from each sample were assigned as an OTU with 97% sequence identity and a dendrogram was created using the dissimilarity matrix based on the Yue & Clayton coefficient [33]. Based on the same dissimilarity matrix, the Unweighted Pair Group Method (UPGMA) was used to cluster all OTUs. Thus, the dissimilarity between multiple samples can be described and the microbial communities from each sample can be analyzed in this context.

Additionally, PCoA plots and Venn diagrams were created to describe whether communities of analyzed samples exhibit the same structure. Rarefaction curves [34] were generated with a 3% sequence dissimilarity cutoff value. We also tested for differences among three cheese types (Cheese A, B and C) and three ripening times (0, 45 and 90d). Significant differences between the two systems were evaluated with the multivariate analysis of variance (Adonis, R project software, http://www.r-project.org). Data collected at two farms were included as a subplot factor in a split plot analysis.

## Results

### T-RFLP analysis of cheese bacterial communities based on 16S rRNA gene amplicons

**Figure 1:**
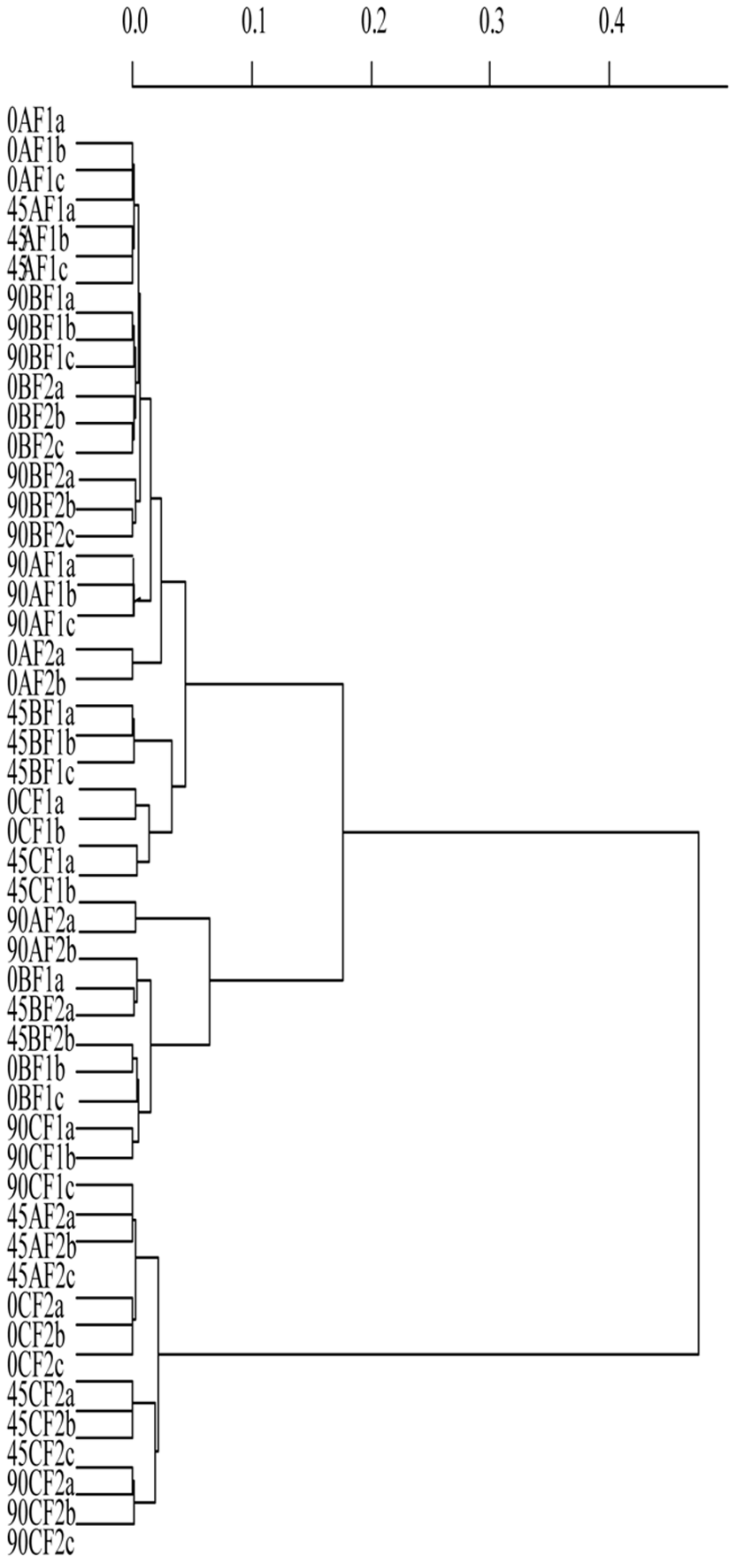
UPGMA dendrogram generated from T-RFLP profiles based on 16S rRNA gene amplicons after direct DNA extraction and PCR amplification throughout the ripening period (0, 45 and 90d) obtained from three Croatian raw ewe's milk cheeses (Cheese A, B and C) from two production sites (F1 and F2). The scale indicates the distance level. Triplicate samples were analyzed (a, b, c).

The T-RFLP analysis was applied to preliminary characterize bacterial communities of three Croatian raw ewe's milk cheese types from two different production sites at different time points of ripening and to screen for the variability between the replicates (Figure 1). In average, 15 T-RFs with heights above 1% of the total height of all peaks present in the electropherogram were observed. T-RFs lengths ranged from 130 bp to 591 bp. The relative abundance of all T-RFs in the electropherograms was from 1.1% to 97.45%. Most peaks were shared by all three cheese types but varied in height. Two T-RFs, with a size of 130 and 498 bp, were dominant (one or both) in all three cheese types at all stages of cheese ripening. When total bacterial community profiles were compared, samples from Cheese C and Cheese A (45d) from F2 were separated from the others, forming a distinct cluster (cluster III) in the UPGMA dendrogram. Other cheese samples formed cluster I and II and showed similar profiles (>98% similar to each other). This indicates that mainly for Cheese C the importance of the milk quality and the cheese manufacturing peculiarities for the microbial composition of mature cheeses. Obviously for Cheese B the quality of the milk did not influence the ripening process to a large extend and despite differences in bacterial communities in fresh cheese samples (0d) between F1 and F2, at the final ripening step bacterial communities were comparable. Samples considered as replicates (taken from one cheese at the same ripening stage from the same production unit) clustered well together.

### Bacterial composition and community structure determined by pyrosequencing of 16S rRNA derived amplicons

**Figure 2:**
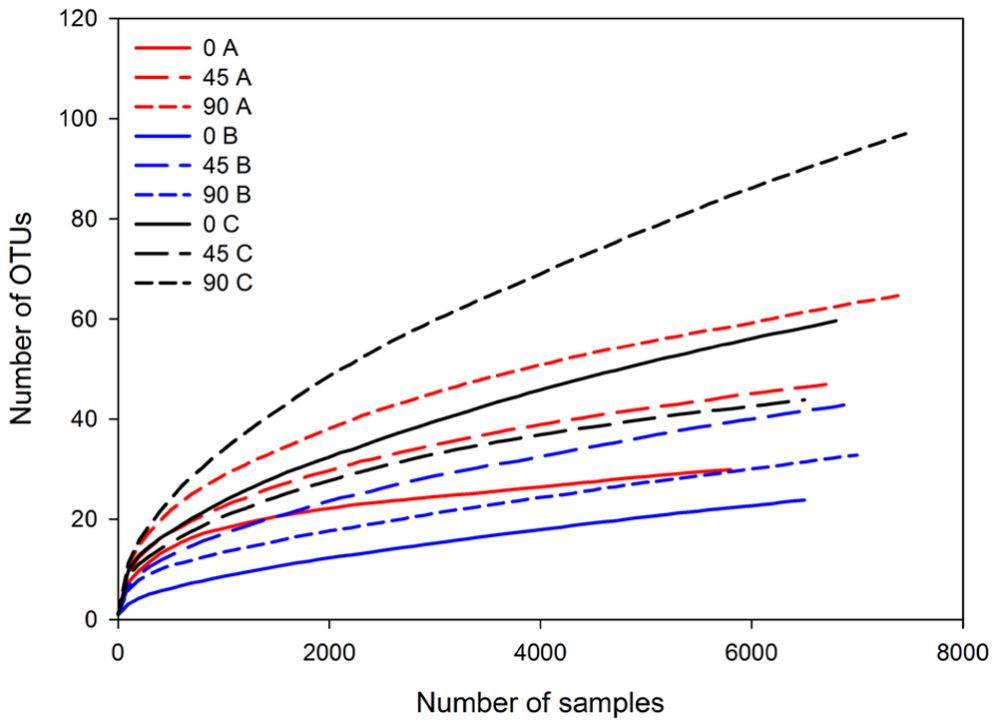
Rarefaction curves of partial sequences of the bacterial 16S rRNA gene after direct DNA extraction and PCR amplification from three Croatian raw ewe's milk cheeses (Cheese A, B and C) throughout the ripening period (0, 45 and 90d) obtained from two production sites (F1 and F2) at a 97% similarity level normalized with respect to sample size. As the most variability were related to F2 only those results are presented.

**Figure 3:**
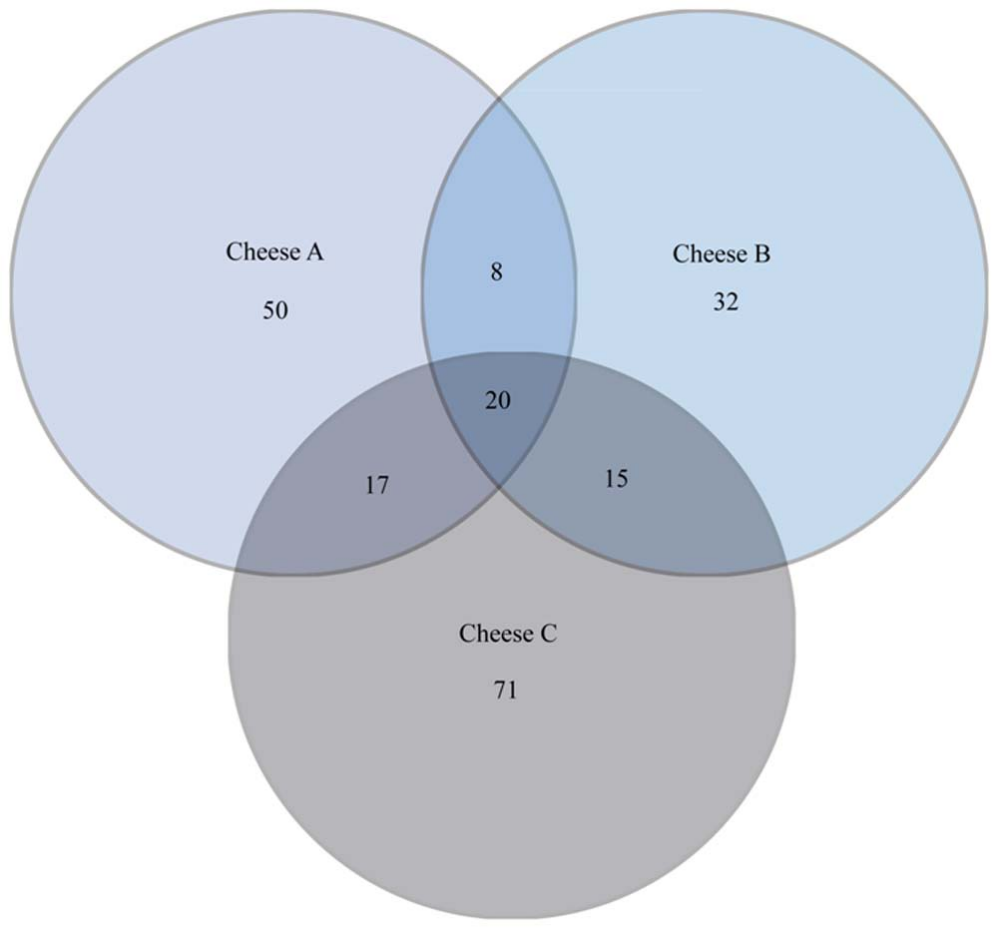
Venn diagram showing the number of specific and common OTUs (OTU_97_) between three Croatian raw ewés milk cheeses (Cheese A, B and C) obtained from two production sites (F1 and F2) throughout the ripening period (0, 45 and 90d) based on partial sequence analysis of the 16S rRNA gene after direct DNA extraction and PCR amplification.

A total of 152296 bacterial raw sequence reads were generated from the PCR amplicons by 454 pyrosequencing. The filtering process removed about 60% of raw bacterial reads, leaving 63629 high-quality bacterial (≥260 bp) reads. After chimera check and removing erroneous reads, a total of 50544 high-quality partial 16S rRNA gene sequences, were obtained, in average 2777 sequences per treatment (cheese type × ripening stage × farm). Rarefaction curves using the dataset generated by the forward primer, normalized by sample size, showed similar patterns for all samples and suggested that major OTU_97_ were covered, except for Cheese C after 90d of ripening (Figure 2). To evaluate the distribution of the obtained 213 OTUs among the different cheese types, a Venn diagram was constructed (Figure 3). The data indicated that 20 OTUs, including 96.48% of the reads, were common to all three cheese types. These results nicely correlate with the very similar T-RFLP pattern obtained from 16S rRNA amplicons mainly for Cheese A and B. The highest number of unique OTUs was noticed for Cheese C (n =  71) and the lowest number of OTUs was noticed for Cheese B (n =  32), confirming the data of the T-RFLP based cluster analysis. Similar results were obtained when the dataset generated by the reversed primer was analyzed (data not shown).

The annotated reads could be grouped into three major phyla: *Firmicutes, Actinobacteria*, and *Proteobacteria*. *Firmicutes* were the most abundant phylums of these, and were dominated by members of the class *Bacilli* belonging to the order *Lactobacillales*. Four families were found among the sequences belonging to this order: *Enterococcaceae, Streptococcaceae, Leuconostocaceae* and *Lactobacillaceae*. The family *Streptococcaceae* was predominant in all three cheese types, and was represented by two genera, *Lactococcus* and *Streptococcus* of which species *Lactococcus lactis* accounted for 54.85% and *Streptococcus parauberis* for 5.68% of all reads respectively. In the family *Enterococcaceae*, the genus *Enterococcus* represented by *Enterococcus* spp. (*E. faecalis*, *E. faecium* and/or *E. durans*) comprised 20.68% of all sequences identified. The genus *Leuconostoc*, mainly *L. mesenteroides*, accounted for 1.70% of reads. Few sequences were assigned to *Lactobacillaceae* represented with two genera, *Lactobacillus* (0.25%) and *Pediococcus* (0.02%). Among them, *Lb. casei/paracasei, Lb. plantarum. Lb. acidipiscis, Lb. brevis, Lb. amylovorus* and *Pediococcus pentosaceus* were identified.

Most sequences of the phylum *Proteobacterium* belonged to the family *Enterobacteriaceae* which was presented by two genera *E. coli/Shigella flexneri* and *Salmonella* spp. They accounted for 3.88% and 1.34% of the reads respectively. The detected *Staphylococcacaee* sequences included two genera, *Staphylococcus* and *Macrococcus*. Among them the species *S. saprophyticus* and *M.* caseolyticus were found to be most abundant, they were present in 1.56% and 2.33% of all reads respectively.

Other bacteria, namely Bifidobacterium thermofilium, Brevibacterium spp. Corynebacterium variabile, Corynebacterium casei, Clostridium tertium, Clostridium perfringens, Macrococcus caseolyticus, Staphylococcus epidermis, Staphylococcus chromogenes, Staphylococcus equorum, Staphylococcus haemolyticus, Thermoanerobacterium thermosaccharolyticum, E. coli, Hafnia alvei, Pseudomonas spp., Janthinobacterium spp., Petrotoga spp., Kosmotoga spp., Megasphaera elsdenii, Mannheimia glucosidal, Mannheimia hemolytica, Str. pluranimaliuma, Str. galollyticus, Leuconostoc citreum, Aerococcus viridans, Vagococcus acidifermentas, Weissella hellenica, Weissella paramesenteroides, Lactococcus raffinolactis and Lactococcus garviae were detected occasionally in some cheese samples comprising less than 0.65% of the total sequences reads.

More than 5% of all sequences were not assigned to genus level or were assigned as unclassified sequences (2.55%). These sequences belong to the families *Enterobacteriaceae* (2.57%), *Enterococcaceae* (0.20%), *Ruminococcaceae* (0.008%) and *Lactobacilliaceae* (0.008%) (Table 1).

**Table 1:** Relative abundance (%) of bacterial species OTUs (OTU_97_) during different time points of ripening (0d, 45d, 90d) of three different Croatian raw ewe's milk cheeses (Cheese A, B and C) obtained from two different farms (F1 and F2) based on partial sequencing of the 16S rRNA gene after direct DNA extraction and PCR amplification. “n” indicates the number of analyzed reads.

Taxon name	% of OTUs																	
	Cheese A						Cheese B						Cheese C					
	F1			F2			F1			F2			F1			F2		
	n = 8424			n = 8415			n = 8424			n = 8424			n = 8424			n = 8424		
	0d	45d	90d	0d	45d	90d	0d	45d	90d	0d	45d	90d	0d	45d	90d	0d	45d	90d
Lactococcus.lactis *	97.32	91.80	86.47	0	0.32	3.89	95.55	98.79	99.22	98.67	64.68	83.37	9.40.	39.52	11.37	33.30	38.07	36.75
Enterococcus spp. **	1.49	5.91	7.05	1.60	45.22	42.65	0.25	0.57	0.32	0.89	0.28	0.39	12.00	31.29	64.60	56.16	55.94	48.22
*Macrococcus caseolyticus*	0.07	0	0.04	0.28	0.14	0.036	1.35	0.10	0.07	0	0	0	39.67	0.13	0.07	0	0	0
*Lactococcus raffinolactis*	0	0	0	0	0	0	0	0	0	0	0	0	0	0	0	0.04	0	0.07
*Leuconostoc citreum*	0	0	0	0	0	0	0	0	0	0	0	0	0	0.10	1.60	0	0.18	0.39
*Leuconostoc mesenteroides*	0	0.10	0	0	2.42	1.39	0	0	0	0.04	1.03	0	0	8.08	14.84	0.14	1.42	1.46
*Pediococcus pentosaceus*	0	0	0	0	0	0	0	0	0	0	0	2.10	0	0	0	0	0	0.04
*Lactobacillus casei/paracasei*	0	0.14	0.21	0	0	0	0	0	0	0	0	0	0	0	0.30	0	0.07	0.07
*Lactobacillus plantarum*	0	0.17	0.07	0	0.17	0.10	0	0	0	0	0	0	0	0	0	0	0	0.04
*Lactobacillus acidipiscis*	0	0	0	0	0.04	0	0	0	0	0	0	2.96	0	0	0	0	0	0
*Lactobacillus brevis*	0	0	0	0	0	0	0	0	0	0	0	0	0	0	0	0	0.07	0
*Lactobacillus amylovorus*	0	0	0.04	0	0	0	0	0	0	0	0	0	0	0	0	0	0	0
*Streptococcus gallolyticus*	0	0	0.04	0	2.18	0	0	0	0.04	0	23.25	5.87	2.85	2.37	1.22	0	0	0
*Streptococcus parauberis*	0	0.25	0.18	13.28	36.64	40.33	0.04	0	0	0.14	0	0	0	0.03	0	6.19	2.70	2.59
*Streptococcus pluranimalium*	0	0	0	0	0	0	0.04	0.04	0.07	0	0.04	0.10	0	0	0	0	0	0
*Bifidobacterium thermophilum*	0	0	0.10	0	0	0	0	0	0.04	0	0	0	0	0	0	0	0	0
*Aerococcus viridans*	0	0	0	0.04	0	0.04	0	0	0	0	0	0	0.07	0	0	0	0	0
*Vagococcus acidifermentas*	0	0	0	0	0	0	0	0	0	0	0	0	0.035	0	0	0	0	0
*Staphylococcus equorum*	0	0	0	0	0.07	0.07	0	0	0	0.10	0.07	0	0.10	0.27	0.53	0	0	0
*Staphylococcu chromogenes*	0.03	0	0	0.36	0.14	0.07	0	0	0	0	0	0	0	0	0	0.03	0	0
*Staphylococcu saprophyticus*	0.14	0	0.93	2.28	2.17	1.14	0.07	0	0.03	0	4.30	2.67	7.54	5.41	0.99	0.32	0.10	0.07
*Staphylococcus sciuri*	0	0	0	0	0	0	0	0.03	0	0	0.39	0.39	0	0	0	0	0	0
*Staphylococcus epidermis*	0.07	0	0	0	0	0	0	0	0	0	0	0	0	0	0	0	0	0
*Corynebacterium casei*	0	0	0.85	0	0	0	0	0	0	0	0	0	0	0	0	0	0	0
*Corynebacterium variabile*	0	0	0.04	0	0	0.06	0	0	0	0	0	0	0	0	0	0	0.04	0.14
*Hafnia alvei*	0.04	0.32	0	0	0	0	0	0	0	0	0	0	0	0	0	0	0	0
*Weissella paramesenteroides*	0	0	0.04	0	0.18	0.57	0	0	0	0	0	0	0	0	0	0	0	0
*Weissella helllenica*	0	0	0	0	0	0	0	0	0	0.04	0	0	0	0	0	0	0	0
*Pseudomonas spp.*	0	0	0	0.53	0	0	0	0	0	0	0	0	0	0	0	0	0	0
*Serratia spp.*	0	0	0	0.39	0	0	0	0	0	0	0	0	0	0	0	0	0	0
*E. coli/Shigella flexneri*	0	0	0	58.98	6.58	3.14	0.04	0	0	0	0.07	0	0.21	0.47	0.34	0	0	0
*Salmonella spp.*	0	0	0	20.44	2.24	0.82	0.07	0.04	0	0	0.10	0	0.10	0.17	0.15	0	0	0
*Mannheimia haemolytica*	0	0	0	0.21	0	0	0	0	0	0	0	0	0	0	0	0	0	0
*Mannheimia glucosida*	0	0	0	0	0	0	0	0	0	0.04	0	0	0	0	0	0	0	0
*Clostridium tertium*	0	0	0	0.04	0	0	0	0	0	0	0	0	0	0	0	0	0	0
*Clostridium perfringens*	0	0	0	0.04	0.07	0	0	0	0	0	0	0	0	0	0	0	0	0
*Brachybacterium spp.*	0	0	0	0	0	0	0	0	0	0	0	0	0	0	0	0	0.07	0.07
*Brevibacterium spp.*	0	0	0.04	0	0	0	0	0	0	0	0	0	0	0	0	0	0	0
*Janthinobacterium spp.*	0	0	0	0.24	0	0	0	0	0	0	0	0	0	0	0	0	0	0
*Kosmotoga spp.*	0	0	0.04	0	0	0	0	0	0	0	0	0	0	0	0	0	0	0
*Petrotoga spp.*	0	0	0.04	0	0	0	0	0	0	0	0	0	0	0	0	0	0	0
*Megasphera elsdenii*	0	0	0.10	0	0	0	0	0	0	0	0	0	0	0	0	0	0	0
*Thermoanaerobacterium spp.*	0.07	0	0.07	0	0	0	0.07	0	0	0	0	0	0	0	0	0	0	0

The contrasting diversity pattern observed in the T-RFLP based analysis for Cheese C could also be confirmed using the sequencing approach. However as the resolution of the sequencing based analysis is much higher compared to the T-RFLP based fingerprinting, differences in response to the production site for the two other cheese types were also visible. Interestingly, for all cheese types the differences in the bacterial community structure were observed not only at the early time points of cheese ripening but also at day 90. Thus the differences were significant at the cheese level (*P*<0.001) as well as at the level of the production unit (*P*<0.05) or either as the combination of both factors (*P*<0.003). Shifts during cheese ripening were only pronounced for selected bacterial species and did not reach significance for the total bacterial community analyzed. For example in Cheese A samples obtained from F1 the prevalence of sequences belonging to lactococci was noticed, whereas in samples from F2 enterococci and streptococci were equally presented except in fresh cheese (0d) which was characterized by strong presence of species belonging to *E. coli/Shigella flexneri* and *Salmonella* spp. Although the number of respective sequences was high in fresh cheeses (58.93 and 20.44%), it decreased below 3.14 and 0.82% respectively after 90d ripening.

**Figure 4:**
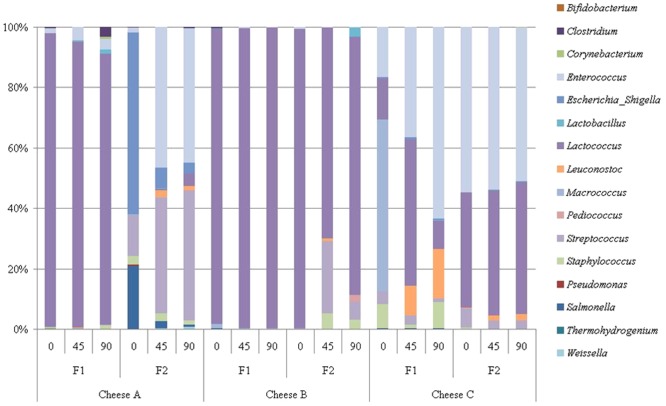
Relative abundance (%) of sequences which could be assigned to genus level (OTU_95_; about 60000 reads in total)) in three Croatian raw ewe's milk cheeses (Cheese A, B and C) based on partial sequence analysis of the 16S rRNA gene gene after direct DNA extraction and PCR amplification. Samples were taken from two production sites (F1 and F2). The numbers on x- axis represents ripening time in days: 0d, 45d and 90d.

**Figure 5:**
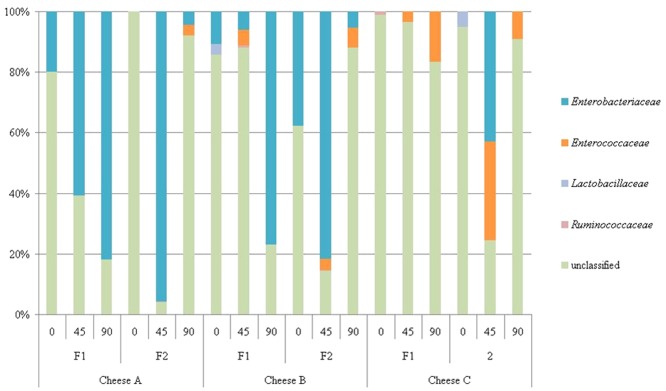
Relative abundance (%) of sequences which could only be assigned to the family level (OTU_90_; about 2000 reads in total) or was assigned as unclassified (about 1200 reads in total in three Croatian raw ewe's milk cheeses (Cheese A, B and C) based on partial sequence analysis of the 16S rRNA gene after direct DNA extraction and PCR amplification. Samples were obtained from two production sites (F1 and F2). The numbers on x- axis represents ripening time in days: 0d, 45d and 90d.

In Cheese C the sequences belonging to *Lactococcus* and *Enterococcus* were predominant. In addition, at F1, a strong presence of *Leuconostoc mesenteroides* (7.53%), *Macrococcus caseolyticus* (13.30%) and *Staphylococcus saprophyticus* (4.62%) was noted. The sequences belonging to *M. caseolyticus* and *S. saprophyticus* were mostly detected in 0d cheese samples (39.67 and 7.54% respectively) and after 90d they declined to 0.07 and 0.99%. The data are summarized in Figure 4 and 5 and Table 1.

## Discussion

A crucial step toward protecting the microbial diversity in artisanal cheese is to investigate and to describe indigenous microbiota in detail during the cheese manufacturing and ripening. The microbial diversity of artisanal cheeses includes a remarkable number of strains which may bring novel characteristics for large-scale industrial application, making it possible to develop new products with new properties. Croatian raw ewe's milk cheeses are farmhouse cheeses made by traditional techniques without the application of a starter culture. In the present study T-RFLP and 454 pyrosequencing of tagged 16S rRNA gene amplicons were used to obtain a more complete overview on the bacterial community structure of Croatian raw ewe's milk cheeses. Considerable biodiversity characterizes the investigated cheeses with 213 different OTUs being identified (at 97% similarity level). This surprisingly high biodiversity might be a fact of the used deep sequencing approach, as the numbers of the so far described species based on cultivation or low resolution molecular fingerprinting methods were much lower [17], [35], [36]. The presented data also nicely points out the importance of the raw ewe's milk and manufacturing peculiarities for the composition of indigenous cheese microflora. In our study fingerprinting just allowed a clear separation between Cheese C from F2 and the other cheese types. Based on the sequencing data it became obvious that this cheese harbors different microbial communities to a larger extent than the other cheese types. Finer differences in the bacterial community structure, as revealed by the sequence analysis between Cheese A and B, or between the two production sites or during the ripening process, could not be resolved by T-RFLP analysis. Besides the quality of the raw ewe's milk the other production factors may play very important roles during cheese ripening. For example, the development of the “secondary microbiota” is mostly influenced by the peculiarities of cheese making and different microclimatic conditions [3], [37] which may explain the differences found in the composition of the microflora in our study.

As expected in many cheeses and dairy products [2], [35], [38], [39] the microbial compositions of Croatian artisanal cheeses were dominated by lactic acid bacteria (LAB). According to the frequencies of reads we could differentiate the LAB populations of Croatian raw ewe's milk cheeses into three groups. The first group is composed of dominant bacteria, namely *Lactococcus lactis* and *Enterococcus* spp. (*E. faecium, E. faecalis* and/or *E. durans*) which were detected in 54.85% and 20.68% of all reads respectively. This group of LAB was also found to be a dominant population in Istrian cheese when investigated by low resolution fingerprinting of the 16S rRNA gene and CFU analysis [23]. The second group of frequently encountered LAB bacteria belonged to *Streptococcus parauberis* and *Leuconostoc mesenteroides* which were identified in 5.68% and 1.70% of total reads, respectively. The third group consists of rare sequences which were detected occasionally comprising less than 0.65% of the total sequences reads (e.g. *Lb. casei/paracasei, Lb. plantarum, Lb. brevis, Lb. amylovors, Lb. acidipiscis, Pediococcus pentosaceus*, *Str. pluranimaliuma*, *Str. galollyticus*, *Leuconostoc citreum*, *Aerococcus viridans, Vagococcus acidifermentas, Weissella hellenica, Weissella paramesenteroides, Lactococcus raffinolactis* and *Lactococcus garviae*).

The LAB groups detected in our study have a pivotal role in a formation of typical organoleptic properties of many artisanal cheeses [3]. They contribute to the specific and unique aroma and taste of many cheese varieties by their unique metabolism of fatty and amino acids turnover [40], [41], [42], [43] and are known for antimicrobial effect due to their ability to produce organic acids and bacteriocins of which nisin from *L. lactis* being the best known and characterized [44]. Mainly the first group of bacteria may play a main role in the fermentation and organoleptic properties of Croatian raw ewe's milk cheeses because of its presence in all tested samples. Although the presence of enterococci in dairy products has long been considered to be an indicator of inappropriate sanitary conditions during milk production and processing, they are considered nowadays to be a normal part of cheese microbiota [45]. In contrast to that, the presence of *Str. parauberis* strains is not desirable as it has been often associated with subclinical and clinical mastitis [46]. However, the high prevalence of *Str. parauberis* was only noticed in Cheese A at F2 and was not associated with other cheese types.

The sequences of non LAB were mostly related to two families, *Enterobacteriacea* and *Staphylococcacae* (5.22 and 3.89% of all reads respectively). The rarely present non LAB (<0.65%) included sequences belonging to *Bifidobacterium thermofilum*, *Brevibacterium* spp., *Corynebacterium variabile, Corynebacterium casei, Clostridium tertium, Clostridium perfringens, Macrococcus caseolyticus, Staphylococcus epidermis, Staphylococcus chromogenes, Staphylococcus equorum, Staphylococcus haemolyticus, Thermoanerobacterium thermosaccharolyticum, E. coli, Hafnia alvei, Pseudomonas* spp., *Janthinobacterium* spp., *Petrotoga* spp., *Kosmotoga* spp., *Megasphaera elsdenii, Mannheimia glucosida* and *Mannheimia hemolytica*. Most of the non LAB genera identified in this study were also frequently detected in other artisanal dairy products. Whereas some authors suggested that they cause milk and cheese spoilage [38], [47], other publications indicate that they may have a secondary activity in the fermentation process of cheeses and can contribute to their taste and flavour [48], [49]. *M. caseolyticus* and *Hafnia alvei* are known for their positive effects to the flavor development. *M. caseolyticus* is involved in casein breakdowns, thus contributing to the formation of aroma precursors as small peptides and free amino acids [50] and *Hafnia alvei* being involved in the accumulation of volatile sulfur compounds [51]. However, the ability of *M. caseolyticus* and *Hafnia alvei* to survive during the maturation of Croatian raw milk cheeses is poor as sequences related to both species were only detected in fresh cheese samples, thus indicating its low ability to adapt to stressful cheese micro conditions.

Although large diversity of potentially pathogenic or spoilage phylotypes were found in milk and fresh Istrian cheese, the bacteriological quality of the ripened products was satisfied such no pathogenic or spoilage microflora were detectable at the end of aging process [52]. Most pronounced were these effects for *E. coli/Shigella flexneri*. These species dominated mainly in fresh samples of cheese A from Farm 2, however their abundance was low, at the end of the ripening process, indicating that these cheese types do not bear risks for consumers in response to contamination with the related microbial species. The similar trend was noticed for *Staphylococcus saprophyticus, E. coli/Shigella flexneri* and *Salmonella* spp., the most frequently detected non LAB species in this study with potential pathogenic activity. These observations indicate the importance of the duration of the ripening process for 90 days to avoid problems related to the hygienic status of the chhese. Similar observations were also made in a previous studies [52], [23]. Several studies indicate that the bacterial succession during cheese maturation depends on their adaptation to stress conditions such as high salt concentrations and low water activity [4] and/or competitive interactions among the microflora [53], [54]. It appears that many of the non LAB species detected in the fresh Croatian raw ewes milk cheeses disappeared during the cheese ripening, thus suggesting the effectiveness of established cheese micro conditions in the prevention of food spoilage independent from the composition of bacterial communities in the basic raw materials.

Of human pathogens, the most concern in food production is related to the genus *Staphylococcus*, especially *S. aureus* due to the pathogenic potential of some strains. Our results indicated the sporadic presence of coagulase negative *S. saprophyticus, S. epidermis, S. chromogenes* and *S. equorum* without occurrence of *S. aureus*. Although some of the *Staphylococcus* species have been recognized as causative agent of urinary infection (e.g. *S. saprophyticus*) or have been proposed as a common cause of subclinical mastitis (e.g. *S. epidermis and S. chromogenes*) [55], [56], their high abundance in raw milk and raw milk cheeses [4], [36], [57] has led to the consideration that several *Staphylococcus* species may be of importance for the specific flavor development of cheese during ripening.

*Clostridium perfringens* is a bacterium detected in Croatian raw milk cheeses that could be of special health concern. *Clostridium perfringens* is widespread in the environment and can contaminate dairy products. Spores have been reported in raw milk and cheese but there have been few reports of illness associated with this bacterium in milk [58]. However, *Clostridium perfringens* was detected in only two fresh cheese samples in this study in a low number of sequences (n = 3) indicating either that good sanitation procedure was applied during Croatian cheese production or that *C. perfrigens* is not well adapted to changing abiotic conditions during the aging of Croatian raw ewe's milk cheeses [59].

The manufacturing of Croatian raw milk cheese involved the covering of fresh cheese in coarse salt. Many of the identified bacterial species are well adapted to the high salt environment and could be recognized as partially or totally salt-tolerant bacteria. Those bacteria include; *Lactococcus lactis*, *Leuconostoc mesenteroides*, *Enterococcus* spp., *Lactobacillus acidipiscis, Staphylococcus saprophyticus*, *Aerococcus viridans, Janthinobacterium* spp. and *Corynebacterium* spp. *which* are part of a dominant or minor microflora of Croatian raw ewe's milk cheeses. The presence of halotolerant bacteria and the adaptation of primary and secondary cheese microbiota to an elevated salt concentration is common for artisanal cheeses [35], [60]. For some species the transfer from the marine environments to the cheeses via sea salt is suggested [61] and their influence on the cheese aroma formation is well documented [62].

Overall our study clearly indicates the benefits of using deep sequencing for the barcoding of bacterial community structures during cheese ripening. The detailed analysis of microbes (not only bacteria count in this respect) in the process of cheese production will without doubt help to stabilize the production process of cheese types where no starter cultures have been used in terms of product quality as well as consumer safety. Future research however might address more specific questions related to the technological or pathogenic potential of microbial communities during cheese ripening based on the analysis of specific genetic markers. In this respect also the role of the cheese quality during ripening (e.g. the water content) for the dynamics of these communities must be taken more into account to deviate best practice protocols for the ripening process.
